# C5a Receptor Deficiency Alters Energy Utilization and Fat Storage

**DOI:** 10.1371/journal.pone.0062531

**Published:** 2013-05-07

**Authors:** Christian Roy, Abhishek Gupta, Alexandre Fisette, Marc Lapointe, Pegah Poursharifi, Denis Richard, HuiLing Lu, Bao Lu, Norma Gerard, Craig Gerard, Katherine Cianflone

**Affiliations:** 1 Centre de Recherche Institut Universitaire de Cardiologie & Pneumologie de Québec, Université Laval, Québec, Québec, Canada; 2 Ina Sue Perlmutter Lab, Children’s Hospital, Harvard Medical School, Boston, Massachusetts United States of America; Wageningen University, The Netherlands

## Abstract

**Objective:**

To investigate the impact of whole body C5a receptor (C5aR) deficiency on energy metabolism and fat storage.

**Design:**

Male wildtype (WT) and C5aR knockout (C5aRKO) mice were fed a low fat (CHOW) or a high fat high sucrose diet-induced obesity (DIO) diet for 14 weeks. Body weight and food intake were measured weekly. Indirect calorimetry, dietary fatload clearance, insulin and glucose tolerance tests were also evaluated. Liver, muscle and adipose tissue mRNA gene expression were measured by RT-PCR.

**Results:**

At week one and 12, C5aRKO mice on DIO had increased oxygen consumption. After 12 weeks, although food intake was comparable, C5aRKO mice had lower body weight (−7% CHOW, −12% DIO) as well as smaller gonadal (−38% CHOW, −36% DIO) and inguinal (−29% CHOW, −30% DIO) fat pads than their WT counterparts. Conversely, in WT mice, C5aR was upregulated in DIO vs CHOW diets in gonadal adipose tissue, muscle and liver, while C5L2 mRNA expression was lower in C5aRKO on both diet. Furthermore, blood analysis showed lower plasma triglyceride and non-esterified fatty acid levels in both C5aRKO groups, with faster postprandial triglyceride clearance after a fatload. Additionally, C5aRKO mice showed lower CD36 expression in gonadal and muscle on both diets, while DGAT1 expression was higher in gonadal (CHOW) and liver (CHOW and DIO) and PPAR**γ** was increased in muscle and liver.

**Conclusion:**

These observations point towards a role (either direct or indirect) for C5aR in energy expenditure and fat storage, suggesting a dual role for C5aR in metabolism as well as in immunity.

## Introduction

It is now widely accepted that obesity is regarded as a state of chronic low-level inflammation, with increased circulating levels of inflammatory markers. Further, white adipose tissue (WAT) is a key endocrine and secretory organ, which produces and releases a wide range of proteins and factors designated adipokines, a number of which are involved in inflammation and the inflammatory response, including leptin, adiponectin, interleukin 1β (IL-1β), IL-6 and monocyte chemotactic protein-1 (MCP-1). The extensive crosstalk between WAT and other tissues, coupled to increased production of inflammation-related adipokines in obesity, is considered important in the development of obesity-associated diseases, predominantly type II diabetes and metabolic syndrome [1]. Tumor Necrosis Factor α (TNFα) is a well-known example of this dual role, as it was originally named ‘cachectin’ due to its proinflammatory effects which are also associated with metabolic effects such as cachexia, weight loss, tissue fatty acid release and insulin resistance [2]. Likewise, IL-6 is an immune-modulating cytokine that influences insulin resistance in peripheral tissues; while toll-like receptors (TLRs), important in immune function, also influence metabolism via fatty acid interactions [3].

The complement system proteins, such as C3 and its cleavage products, C3a and C3adesArg (acylation stimulating protein, ASP), as well as factor B, adipsin (factor D) and others, are additional examples of immune system proteins present in the WAT environment, further expanding this concept of a dual immune-metabolic role in adipose tissue [4], [5]. In fact, this association between the complement system and WAT has a storied past, as shown by the early discovery that acquired lipodystrophy was found to coincide with C3 hypocomplementemia [6], even prior to the first descriptions of familial C3 deficiency presenting with partial lipodystrophy [7]. ASP/C3adesArg has been shown to stimulate triglyceride synthesis in adipocytes by promoting fatty acid uptake and glucose transport while C3^−/−^ mice, which are obligately deficient in ASP, as well as factor B^−/−^ and adipsin/factor D^−/−^ mice, all demonstrate both immune and metabolic alterations, including altered energy metabolism and fat clearance [8], [9]. Furthermore, it has recently been shown by Gauvreau *et al* that properdin, a C3 convertase stabilizer, also plays a dual role as demonstrated by decreased energy expenditure and altered postprandial lipid clearance in properdin^−/−^ mice [10].

C3a receptor (C3aR), C5a receptor (C5aR) and C5L2 (G-protein receptor 77, GPR77) constitute a trio of related receptors which variously bind the anaphylatoxins C3a, C3adesArg/ASP, C5a and C5adesArg, all cleavage products of complement pathway activation [11]. While C3aR binds only C3a, but not the desarginated peptide C3adesArg/ASP, C5aR binds both C5a and (to a lesser extent) C5adesArg. C5L2 ligand binding remains controversial: while both C5a and C5adesArg bind with high affinity, the functional outcome (signalling vs. decoy receptor) remains unclear; further some [12], [13] but not all [14], [15] studies report low affinity binding of C3adesArg/ASP and C3a to C5L2.

While all three receptors appear to have well-defined immune-related roles, the potential duality of these receptors in adipose tissue and in immune-metabolic function is only beginning to be considered [16]. In this respect, several studies have demonstrated a role for C3a and C3aR in asthma, sepsis, liver regeneration, as well as autoimmune encephalomyelitis [17], [18], and only just recently, a role for C3aR in insulin resistance and adipose tissue macrophage infiltration has been proposed [19]. Similarly, studies with C5L2^−/−^ mice have demonstrated roles for C5L2 in inflammation [20], [21] and sepsis [22] as well as insulin resistance and lipid metabolism [23], [24].

C5a has multiple immune functions, including stimulation of histamine secretion in mast cells, chemotactic activity, and facilitation of phagocyte mobilization. However, in addition to myeloid cells and tissues, C5aR is also widely expressed in connective tissue, circulatory system, kidney, liver, lung and skin [25] and is recognized as a pleiotropic factor with a range of biological functions such as contraction of smooth muscle and increasing capillary vessel permeability [26], [27], [25]. C5aR deficient mice show alterations in many of the disease processes that involve C5a, such as mucosal defence, rheumatoid arthritis, contact sensitivity, glomerulonephritis, pulmonary hypersensitivity, peritonitis, and sepsis [25], [28]. While C5a-C5aR often plays a protective role, excess generation can lead to negative consequences and antagonists have been developed for use in disease models [25], [28].

C5a and its receptor are also present in the central nervous system [29] and it has been reported that C5a stimulates food intake after central administration [30]. Further, a very recent study in rats by Lim et al [31], demonstrated that C3a and C5a could both affect adipocyte nutrient storage, and *in vivo* administration of specific C3aR and C5aR antagonists reduced fat tissue size when coupled with a high fat diet. Given the dual immune-metabolic functions of many adipokines, cytokines and complement system proteins, including C3aR and C5L2, we hypothesized that C5aR^−/−^ (C5aRKO) mice would also demonstrate a duality in C5aR function, either through a direct effect or an indirect effect (consequent to changes in immune function due to the absence of C5aR). The aim of the present study was to investigate energy homeostasis, particularly energy expenditure, growth, physical activity, food intake, substrate utilization and storage, and gene expression in relation to adipose, muscle and liver tissues in C5aR deficient mice under normal low fat chow (CHOW) and high fat diet-induced obesity (DIO) conditions.

## Materials and Methods

### Ethical Statement

All protocols were conducted in accordance with the CACC guidelines and were pre-approved by the Laval University Animal Care Committee (Quebec, Canada) and the Laval Hospital Research Institute (CRIUCPQ, Quebec, Canada) committee. All research protocols followed these guidelines and all efforts were made to minimize suffering. At protocol initiation, 8–9 week-old mice were housed individually in a sterile barrier facility with a fade-in/fade-out 12 h light: 12 h dark cycle within the animal facility. The cage also included enrichment toys such as cotton batting and plastic tubes. Mice were euthanized at 22–23 weeks of age after 14 weeks of diet through anesthesia followed by cervical dislocation prior to dissection.

### Mice

The C5aR deficient homozygous mice were kindly provided by John Lambris (University of Pennsylvania School of Medicine, Department of Pathology & Laboratory Medicine Philadelphia, PA) in collaboration with Craig Gerard (Pulmonary Division, Department of Pediatrics, Children’s Hospital, Department of Medicine, Harvard Medical School, Boston, Massachusetts) via Jackson Laboratories (The Jackson Laboratory, Bar Harbor, ME). Detailed methodology for development of these transgenic mice has been previously published elsewhere [32]. Homozygous breeding in our internal colony generated C5aRKO littermates. C57BL6 mice (WT), purchased from Jackson Laboratories were bred in our animal facility and matched for age, sex, and starting body weight with the C5aRKO cohort. At protocol initiation, 8–9 week-old mice were housed individually in a sterile barrier facility with a fade-in/fade-out 12 h light: 12 h dark cycle within the animal facility. The cage also included enrichment toys such as cotton batting and plastic tubes. Mice were placed on low fat chow diet (CHOW: 18% kcal fat, 58% complex carbohydrate (no sucrose), #2918; Harlan Laboratories, Montreal, Canada) or a high fat high sucrose diet-induced obesity diet (DIO: 45% kcal fat, 35% carbohydrate of which half (17% total) is sucrose, #D12451; Research Diets Inc., New Brunswick, NJ, USA) and were euthanized at 22–23 weeks of age after 14 weeks of diet through anesthesia followed by cervical dislocation prior to dissection.

Mice were evenly distributed in separate subset studies for food intake (n = 14 per group) and for metabolic chamber analysis (n = 8 per group). Body weight and food intake were measured twice per week for 14 weeks. Calorimetric measurements were taken at week 1 and 12. Oxygen consumption (VO_2_) and carbon dioxide production (VCO_2_) were measured over a 48-hour period in an open circuit system following 48 hours equilibration as previously described [33]. VO_2_ and VCO_2_ were calculated as mL/kg/min and RQ (respiratory quotient) was taken as the ratio of VCO_2_/VO_2_.

At termination of protocol, mice were fasted for 6 h, euthanized through anesthesia followed by cervical dislocation, blood was collected through cardiac puncture, and tissues were collected and immediately frozen in liquid nitrogen, then transferred to −80°C. All protocols were conducted in accordance with the CACC guidelines and approved by the Laval University Animal Care Committee (Quebec, Canada) and the Laval Hospital Research Institute (CRIUCPQ, Quebec, Canada) committee.

### Plasma Analysis

Blood was collected after 6 h fasting. Blood glucose levels were measured using a glucometer with strips (LifeScan, Milpitas, CA, USA). Plasma triglyceride (TG), non-esterified fatty acid (NEFA) and cholesterol were measured by colorimetric enzymatic kits as follows: plasma TG (Roche Diagnostics, Indianapolis, IN, USA), NEFA and cholesterol (Wako Chemicals, Osaka, Japan). Mouse leptin RIA kits were purchased from Millipore (Linco Research, St-Charles, MO, USA). Plasma insulin was measured using the Ultra Sensitive Mouse Insulin ELISA kit from Crystal Chem Inc. (Downers Grove, IL, USA). Plasma C5a was measured using a sandwich ELISA (DuoSet ELISA, cat DY2150) from R&D Systems (Minneapolis, MN, USA). A mouse complement C3 ELISA kit (Kamiya Biomedical, Seattle, WA, USA) was used to measure total C3, including native and breakdown products, and was used according to the manufacturer’s instructions with calculation by linear regression against a standard curve.

### Indirect Calorimetry

For indirect calorimetry, mice were placed in metabolic chambers for measurement of oxygen consumption (VO_2_), carbon dioxide production (VCO_2_), and respiratory quotient (RQ) over a 48-h period in an open-circuit system with an S-3A1 oxygen analyzer and a CD-3A carbon dioxide analyzer, both from Applied Electrochemistry (Pittsburgh, PA, USA). VO_2_ and VCO_2_ were calculated as millilitres per minute, and respiratory quotient (RQ) was calculated as the ratio of VCO_2_/VO_2_. The metabolic chambers from AccuScan Instruments (Columbus, OH, USA) consisted of rectangular, air-proof cages (30×30×20 cm), linked to an open-circuit, flow-through calorimetric device connected to a computer-controlled system of data acquisition. In these chambers, it was also possible to estimate locomotor activity (via a grid of invisible infrared light beams, XY movement corresponding to distance, XYZ corresponding to total activity), while analysing indirect calorimetry.

### Intragastric Fat Meal Administration, Insulin and Glucose Tolerance Tests

Oral fat load tests, consisting of olive oil (13 µl/g of body weight with 100 µl of air above the oil), were administered by intragastric gavage feeding tube following an overnight fast after 12 weeks of diet (n = 5 mice per group). Tail vein blood samples were taken at 0, 2, 3, 4, and 6 h after administration of the fat load. All blood samples were collected in 2% EDTA, separated by centrifugation at 5,000 *g* for 5 min, and stored at –20°C.

At week 13 of the diet regimen, an insulin tolerance test (ITT) was performed following a 6 h fast (n = 7 mice per group). Blood samples were taken at 0, 15, 30, 60 and 90 min after an intra-peritoneal insulin injection (0.5 mU/g of body weight, Humulin, Lilly). Glucose was measured using a glucometer, as described above.

In a separate group of mice, at week 13, glucose tolerance tests (GTT) were conducted following an overnight fast (n = 7 mice per group). An intraperitoneal glucose injection (2 mg/g of body weight) was given, and blood samples were taken at 0, 15, 30, 60, and 90 min from the tail vein. Glucose was measured using a glucometer, as described above.

### MCP-1, KC, C3 and ASP Secretion in Adipose Tissue

Gonadal adipose tissues from WT and C5aR KO (n = 5–8) CHOW and DIO treated mice were collected, minced, and aliquots were incubated overnight in serum-free medium (DMEM/F12) and used for determination of C3 (as indicated above) and mouse ASP/C3adesArg. ASP was measured using a sandwich ELISA as previously described (Gao 2010). Note, the antibody used in this ELISA is specific for a neoepitope exposed in mouse C3a and its desArginated form (ASP/C3adesArg), but does not cross-react with the native protein C3. Mouse ASP (C3a) ELISA reagents were from BD Pharmingen (Franklin Lakes, NJ, USA). In separate experiments, adipose tissue aliquots were treated with C5a (VWR, Mississauga, ON, Canada), LPS (Sigma-Aldrich, Oakville, ON, Canada), and LPS+C5a for 24 hr. The media was then collected and used in ELISA assays for determination of mouse KC/CXCL1 (Cat # DY453) and mouse MCP-1/CCL2/JE (Cat # DY479) from R&D Systems (Minneapolis, MN, USA).

### RNA Extraction and Real Time qPCR Analysis

RNA was extracted from quadriceps muscle, liver and gonadal fat pad of WT and C5aRKO mice, using RNeasy Plus Universal Mini Kit. For muscle and liver, 0.4 µg of total RNA and for gonadal fat, 1 ug of total RNA were retrotranscribed by RT^2^ First Strand kit (Qiagen Inc., Mississauga, ON, Canada). Genomic DNA contamination was eliminated by DNase treatment included in RT^2^ First Strand kit. RT^2^ SYBR® Green qPCR Master Mix (Qiagen Inc., Mississauga, ON, Canada) was used and a 3-step PCR was performed using CFX96™ Real-Time PCR Detection System (Bio-Rad Laboratories, Mississauga, ON, Canada). C5aR primers were obtained from Qiagen (Gaithersburg, MD). Mouse qPrimerDepot resource site (mouseprimerdepot.nci.nih.gov) was used to identify specific primers for *C5L2, DGAT1, DGAT2, CD36, LPL, FAS, PPARG, F4/80, CD11c* and *Eef2* (as housekeeping gene). Primers were ordered from Alpha-DNA (Montreal, Canada). All sequences for the primers used are listed in Table S1. As the efficiency of the housekeeping gene Eef2 was similar to that of the genes of interest, relative gene expression was calculated and corrected using Eef2 using the delta-delta-Ct method.

### Statistical Analysis

Graphical results are presented as mean ± SEM. Each group contains results from a mean of 8–11 mice per group. Groups were compared by t-tests, one-way or two-way ANOVA or by two-way repeated measures ANOVA (RM-ANOVA) followed by Bonferroni post-test, as appropriate for the data, using Prism 5.0 software (GraphPad Software Inc., La Jolla, CA, USA). Statistical significance was set at P<0.05, where NS indicates not significant.

## Results

### Effect of C5aR Deficiency on Food Intake

Since it has previously been demonstrated that central administration of C5a stimulates food intake [30], food consumption as well as other energy-related parameters were first evaluated in wildtype and C5aR knockout mice on both low fat (CHOW) and high fat (DIO) diets. Importantly, to avoid dominant peer influence, mice were housed individually; food intake was measured twice per week. As shown in Table 1, there was no difference in total calories ingested between KO and WT on either CHOW or DIO diets, although there was the expected increase in caloric intake within each genotype on the DIO diet.

**Table 1:** Body and tissue weights, food intake and plasma parameters in wildtype and C5aR knockout mice.

	WT CHOW	C5aRKO CHOW	WT DIO	C5aRKO DIO
**Initial body weight (g)**	23.1±0.4	22.6±0.4	23.8±0.5	23.1±0.5
**Cummulative food intake (kcal)**	1247±28	1280±25	1505±29^a^	1513±53^b^
**Glucose (mmol/L)**	8.91±1.10	9.36±0.81	10.63±0.75	9.87±0.77
**Insulin (ng/ml)**	0.39±0.03	0.35±0.04	0.53±0.05^a^	0.48±0.07
**Leptin (ng/ml)**	5.46±0.28	5.39±0.75	18.39±3.37^a^	15.87±3.23^b^
**TG (mmol/L)**	0.41±0.02	0.30±0.02*	0.54±0.04^a^	0.37±0.03***
**NEFA (mmol/L)**	0.62±0.04	0.49±0.03*	0.80±0.05^a^	0.62±0.05*
**Cholesterol (mmol/L)**	0.48±0.01	0.42±0.03	0.69±0.04^a^	0.57±0.03*
**TISSUE WEIGHTS**				
**Muscle (g)**	0.32±0.01	0.31±0.01	0.33±0.01	0.31±0.01
**Liver (g)**	1.16±0.03	1.03±0.05*	1.08±0.04	1.03±0.05

Body and tissue weights, food intake and plasma triglyceride (TG), non-esterified fatty acids (NEFA) and cholesterol were measured in wildtype (WT) and C5aRKO on chow and high fat high sucrose diet-induced obesity (DIO) diet for n = 10–14 per group. Results are expressed as means ± SEM, and statistical differences were evaluated by ANOVA followed by Bonferroni post-test, where *P<0.05 and ***P<0.0001 vs WT on their respective diet, or for DIO vs CHOW where “a” indicates P<0.05 for WT DIO vs WT CHOW and “b” indicates P<0.05 for C5aRKO DIO vs C5aRKO CHOW.

### Oxygen Consumption and Locomotor Activity in C5aR Deficient Mice

**Figure 1:**
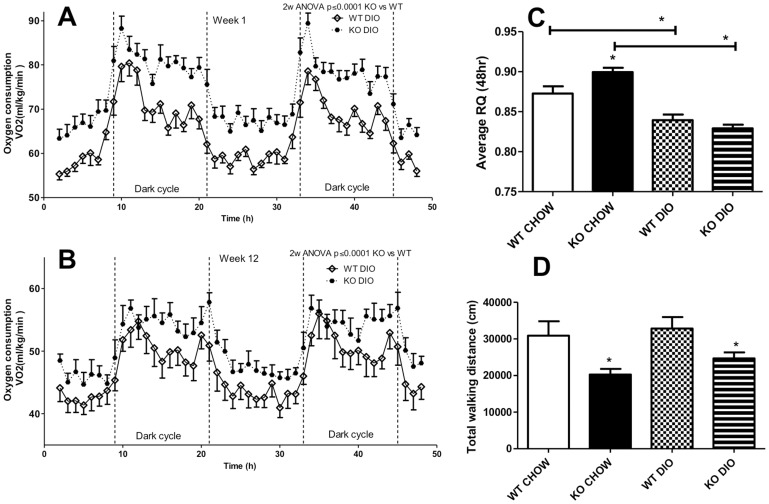
Oxygen consumption and locomotor activity. (A and B): Oxygen consumption after diet treatment for one week (A) and 12 weeks (B), evaluated over a 48 hour period for wildtype (WT, open circles) and C5aRKO mice (solid circles) on a high fat high sucrose diet-induced obesity (DIO) diet. (C): Respiration quotient (RQ) for the same 48 hour period for WT and C5aRKO mice on CHOW or DIO diet. (D): Total walking distance over the 48-hour period for WT and C5aRKO mice on CHOW or DIO diet. Results are presented as means ± SEM of n = 8 mice per group. For calorimetry results, “dark cycle” indicates the active period. Data were analysed by 2-way RM-ANOVA (A and B) and by ANOVA followed by t-test (C and D), where *P<0.05 for C5aRKO vs WT on their respective diets, unless otherwise indicated.

Energy expenditure was measured with indirect calorimetry, using several parameters. After one week of DIO diet, as shown in Fig. 1A, O_2_ consumption was higher in C5aRKO mice compared to WT mice (p<0.001, 2way ANOVA). Over the 48-h period, this increase was present in both the dark cycle, when mice are known to initiate eating, and in the light (sleeping) cycle. There was no significant difference between KO and WT mice on CHOW diet (data not shown). Interestingly, the same results were observed after 12 weeks of the DIO diet, as shown in Fig. 1B, O_2_ consumption was increased over a 48-h period in C5aRKO mice compared to WT mice. Analysis of RQ, which is indicative of relative energy substrate usage (glucose vs. fatty acid), indicates that C5aRKO mice receiving a CHOW diet had a preference for glucose oxidation, as indicated by higher RQ compared with WT mice (Fig. 1C). On a DIO diet, RQ decreases, indicative of greater fat oxidation relative to glucose (Fig. 1C). Physical activity, evaluated as two components: cumulative walking distance and total activity, was also monitored during the time in the calorimetric chambers. Cumulative walking distance represents movement within the x-axis direction, whereas total activity accounts for movement within all three axes. Over the 48-h time period, C5aRKO mice walked less vs WT mice (Fig. 1D) regardless of CHOW or DIO diet, although there was no difference in total physical activity (data not shown).

### Effect of C5aR Deficiency on Diet-induced Obesity

**Figure 2:**
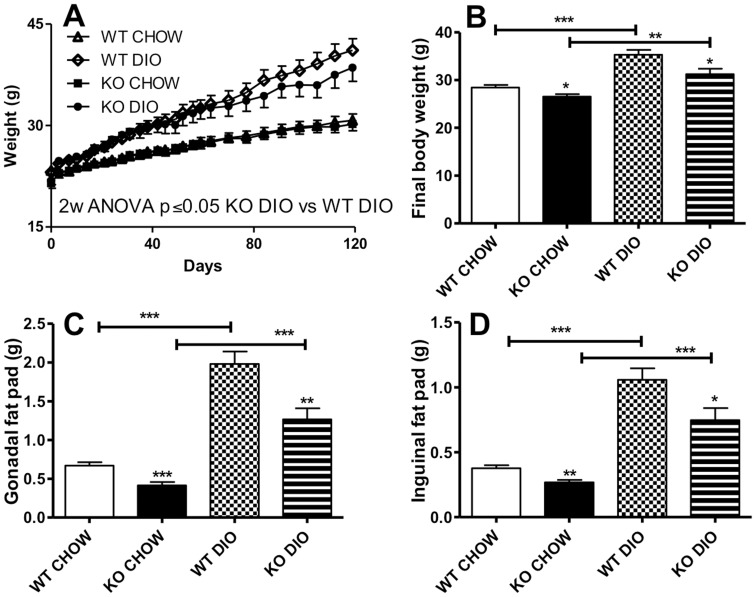
Altered body weight and fat pad weight in C5aR deficient mice. (**A**): Growth curve during diet treatment with CHOW or high fat high sucrose diet-induced obesity (DIO) diet in wildtype (WT) and C5aRKO mice (**B**): Final body weight for WT and C5aRKO mice on CHOW or DIO diet. (C and D): Tissue weights of gonadal (C) and inguinal (D) fat pads of WT and C5aRKO mice at the end of the protocol. Results are presented as means ± SEM of n = 12–14 mice per group, data are analysed by 2 way ANOVA (A), or ANOVA followed by t-test (B–D) where *P<0.05, **P<0.01 and ***P<0.001 and comparisons refer to C5aRKO vs WT on their respective diets, unless otherwise indicated.

At initiation of the dietary protocol, mice were matched for age and initial body weight (Table 1 and Fig. 2A). Over the diet protocol, the C5aRKO mice gained on average 2–4 g (7–12%) less weight than their WT counterparts during the 14 weeks of the study (Fig. 2A and B). This resistance to weight gain observed in the C5aRKO was reflected by smaller gonadal (−36% to −38%) and inguinal fat pads (−29% to −30%) (Fig. 2C and D), with a slight decrease in final liver weight on CHOW diet when compared to WT control (Table 1). On the other hand, while there were DIO diet-induced changes in leptin, an adipokine strongly reflective of adipose tissue mass, there was no difference between C5aRKO and WT mice (Table 1) in spite of differences in adipose tissue mass.

### *C5aR* and *C5L2* Expression in Insulin-sensitive Tissues of Mice

**Figure 3:**
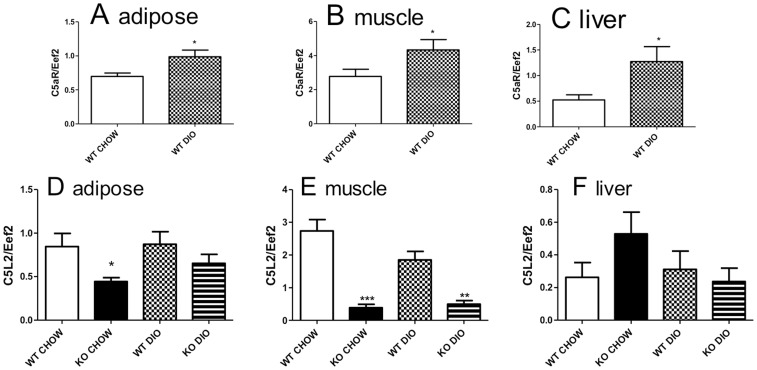
C5aR and C5L2 expression in adipose, muscle and liver of mice. C5aR mRNA expression for WT CHOW (open bars) and WT diet-induced obesity (DIO) (checkered bars) for (A) gonadal adipose tissue, (B) quadriceps muscle, and (C) liver were measured. C5L2 mRNA expression for WT CHOW (open bars), WT DIO (checkered bars), KO CHOW (black bars) and KO DIO (striped bars) for (D) gonadal adipose tissue, (E) quadriceps muscle, and (F) liver were evaluated. Results are expressed as means ± SEM; n = 9–12 per group. Statistical differences were determined by t-test, for C5aRKO vs WT on their respective diets where *P<0.05, **P<0.001 and ***P<0.0001.

Although C5a-C5aR functions have been largely investigated from the perspective of an immune response, based on the detected differences in energy expenditure, physical activity and adipose tissue weights, the results suggested that C5aR might play a role (either direct or indirect) in adipose, muscle and liver metabolism. Accordingly, *C5aR* gene expression was evaluated in the WT mice fed a CHOW or DIO diet. Expression was identified in gonadal adipose, muscle and liver tissues (Fig 3 A, B and C), where DIO diet fed WT mice showed a higher expression of C5aR vs CHOW diet fed WT mice for all three tissues. In addition, *C5L2* expression was also evaluated in WT and C5aRKO mice on both diets (Fig 3 D-F). We observed a striking lowering of *C5L2* expression in the muscle of C5aRKO mice compared to their WT counterpart (Fig 3E), with a smaller effect in adipose tissue (3D), but no difference in *C5L2* expression in liver tissue (Fig 3F).

### Metabolic Insulin and Glucose Response of C5aRKO Mice

**Figure 4:**
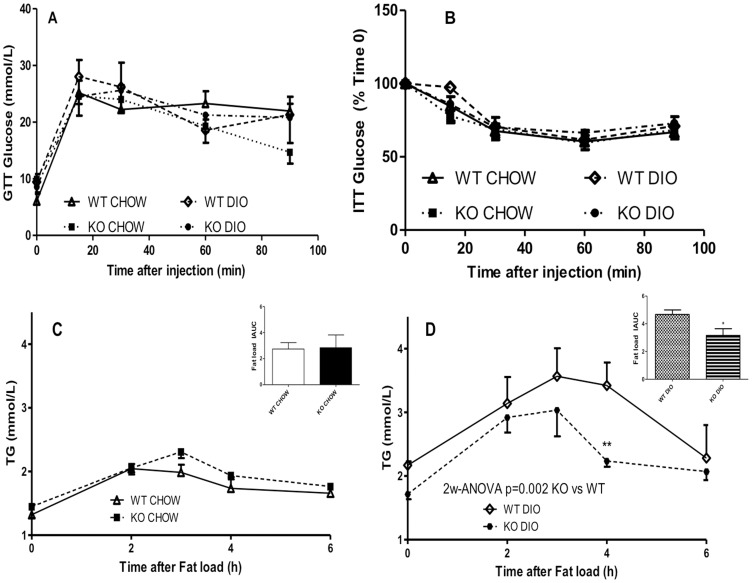
Metabolic response to glucose and insulin tolerance tests and postprandial lipid clearance in C5aRKO mice. (A): Plasma glucose levels following an intraperitoneal injection of glucose in WT (open symbols) and C5aRKO (solid symbols) mice after 13 weeks of low fat CHOW or diet-induced obesity (DIO) diet for n = 6–7 mice per group. (B): Plasma glucose levels following an intra-peritoneal injection of insulin in WT (open symbols) and C5aRKO (solid symbols) mice after 13 weeks of CHOW or DIO diet for n = 6–7 mice per group. Triglyceride (TG; C and D) postprandial lipid clearance following an oral fat load for WT (open symbols), C5aRKO (solid symbols) mice on CHOW (C) and diet-induced obesity (DIO) (D) diets for n = 5 mice per group. Results are presented as means ± SEM; data are analysed by 2-way RM-ANOVA followed by Bonferroni post-test for individual time point differences, where *P<0.05, and **P<0.001 for KO vs. WT and by t-test for the incremental area-under-the curve (IAUC), where *P<0.05 vs WT on the same diet.

Insulin-related metabolism was further evaluated in detail under fasting conditions, as well as following insulin sensitivity tests. Fasting blood glucose levels were similar in all 4 groups and while fasting blood insulin levels were identical between C5aRKO and the WT mice on the same diet, the WT mice on the DIO diet had increased insulin levels as compared to their CHOW counterparts (Table 1). At week 13, both glucose and insulin tolerance tests were administered to assess insulin sensitivity on both CHOW and DIO diets (Fig. 4 A and B). In spite of the decreased body and adipose tissue weights, blood glucose excursion in both groups of C5aRKO mice was not different during the glucose tolerance test (GTT) (Fig. 4A). Additionally, no significant difference in the insulin-induced decrease in blood glucose during the insulin tolerance test (ITT) was detected in the C5aRKO mice on either diet compared with WT mice (Fig. 4B). Furthermore, there was no difference in *IRS1* expression in adipose, skeletal muscle and liver tissues (data not shown).

### C5aRKO Lipid Metabolism

In addition to assessment of adipose tissue depot weights (Fig 2), *in vivo* lipid metabolism and several lipid parameters were also analyzed. At the end of the diet protocol, fasting plasma triglyceride, NEFA and total cholesterol were measured. As indicated in Table 1, C5aRKO mice on the CHOW diet showed lower plasma levels of triglyceride and NEFA as compared to WT. Similarly on DIO diet, C5aRKO mice had lower plasma levels of triglyceride, NEFA and total cholesterol vs WT counterparts. Although there was no difference between WT and C5aRKO mice in postprandial fat tolerance clearance on a CHOW diet, C5aRKO mice demonstrated a significantly more rapid clearance on the DIO diet (Fig 4C and D). To evaluate the mechanism for these changes in both fasting and postprandial lipids in C5arKO mice, related plasma variables, as well as muscle, liver and adipose tissue parameters were evaluated.

**Figure 5:**
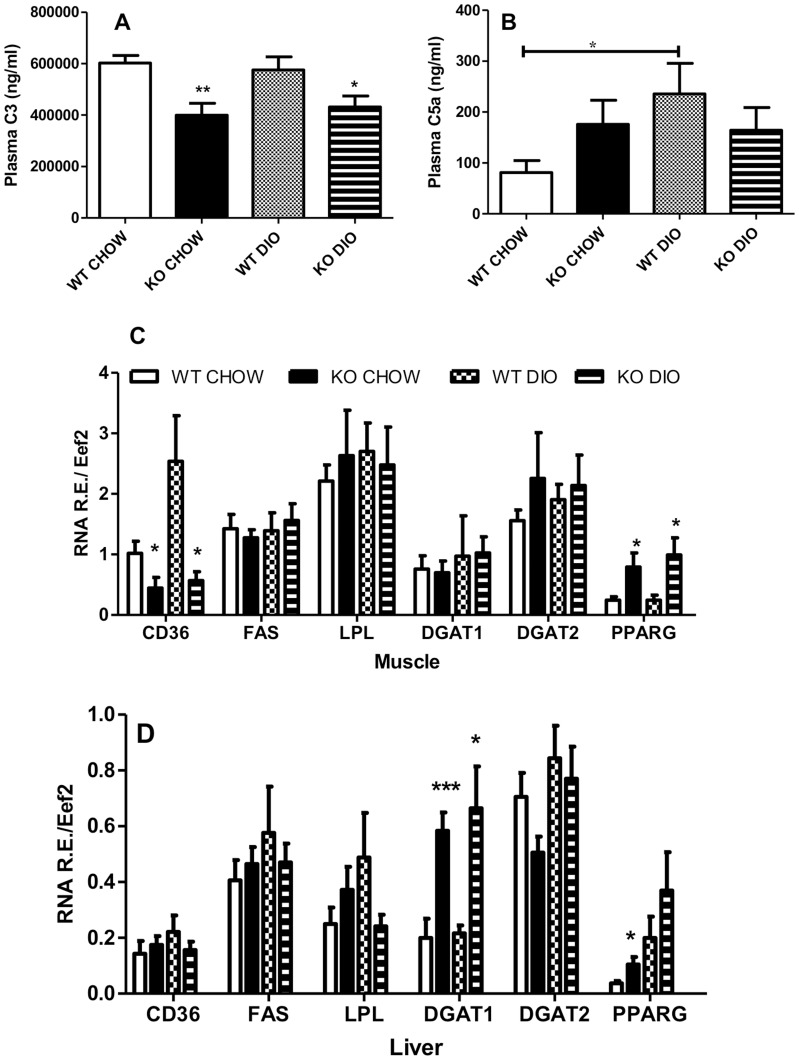
C3 and C5a plasma level and mRNA expression in muscle and liver of genes implicated in substrate utilization. Plasma levels of C3 (A) and C5a (B) in WT and C5aRKO on chow and DIO diet for n = 8–12 per group. Gene expression in quadriceps muscle tissue (C) and liver (D) for fatty acid transporter (*CD36*), fatty acid synthase (*FAS*), lipoprotein lipase (*LPL*), diacylglycerolacyltransferase-1 (*DGAT1*), diacylglycerolacyltransferase-2 (*DGAT2*) and peroxisome-proliferator activated receptor gamma (*PPARG*) were measured in WT CHOW diet (open bars), C5aRKO CHOW (solid bars), WT diet-induced obesity (DIO) diet (checkered bars) and C5aRKO DIO (striped bars) for n = 6–12 per group. Results are expressed as means ± SEM; statistical differences were determined by t-test, where *P<0.05 and**P<0.001 and ***P<0.0001 for C5aRKO vs WT on the same diet, unless otherwise indicated.

While there was no change in circulating ASP (data not shown), C3 plasma levels were lower in C5aRKO mice when compared to their WT counterparts (Fig 5A), while C5a plasma level was influenced by the DIO diet only in the WT group (Fig. 5 B).

Additionally, mRNA expression of key parameters in lipid metabolism was evaluated in skeletal muscle and liver. No differences were seen in *LPL, FAS, DGAT1* and *DGAT2* in muscle. There were however marked differences identified in skeletal muscle such as higher *PPARG* expression and lower *CD36* expression in C5aRKO mice on the CHOW and DIO diet (Fig. 5C). In liver, there were no differences in *CD36, FAS* and i. However, there was an increase in *PPARG* (Fig. 5D). In liver, DGAT2 is more closely associated with esterification of de novo synthesized fatty acids, whereas DGAT1 is more associated with esterification of exogenously derived fatty acids [34] and interestingly, C5aRKO mice demonstrated higher levels of *DGAT1*, with reciprocal decreases in *DGAT2* expression (Fig 5D).

### C5aRKO Adipose Tissue Function

**Figure 6:**
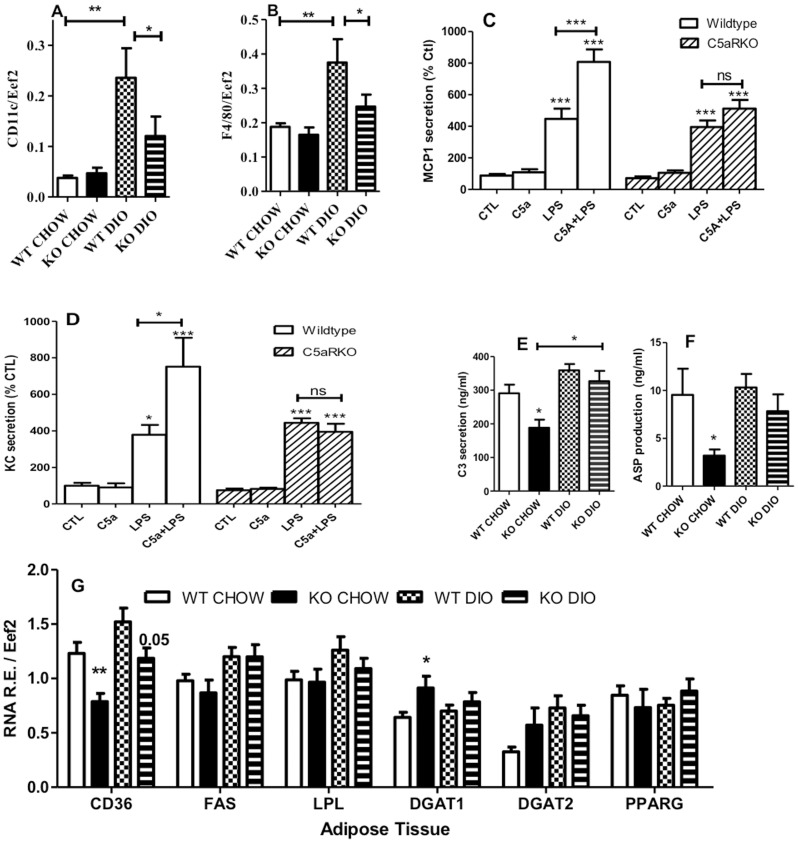
Evaluation of adipose tissue immune and metabolic function in C5aRKO mice. Gene expression for immune factors *CD11c* (A) and *F4/80* (B) and for lipid-related factors fatty acid transporter (*CD36*), fatty acid synthase (*FAS*), lipoprotein lipase (*LPL*), diacylglycerolacyltransferase-1 (*DGAT1*), diacylglycerolacyltransferase-2 (*DGAT2*) and peroxisome-proliferator activated receptor gamma (*PPARG*) (G) were evaluated in WT CHOW diet (open bars), C5aRKO CHOW (solid bars), WT DIO diet (checkered bars) and C5aRKO DIO (striped bars) in gonadal adipose tissue, where n = 6–12 per group. MCP-1 (C) and KC (D) secretion were measured in *ex vivo* adipose tissue in WT (white bars) and C5aRKO (diagonally striped bars) mice following overnight (24h) treatment with C5a (50 ng/mL), LPS, or both vs control (CTL) where n = 8–10 per group. *Ex vivo* C3 secretion (E) and ASP production (F) were measured in media from adipose tissue of WT CHOW diet, C5aRKO CHOW, WT DIO and C5aRKO DIO incubated for 24 h. Results are expressed as means ± SEM; statistical differences were determined by ANOVA followed by Bonferroni post-test, where *P<0.05, **P<0.001 and ***P<0.0001 vs CTL (C, D) or vs WT on their respective diet (A,B,E-G) unless otherwise indicated.

As noted above, C5aRKO mice have smaller adipose tissue depots (Fig 2C and D) and, coupled to the higher C5aR expression level with DIO diet (Fig 3A), this suggests potential alterations in adipose immune function and macrophage infiltration. Accordingly, macrophage markers *F4/80* and *CD11c* were evaluated in adipose tissue. DIO treatment resulted in higher *F4/80* (Fig 6A) and *CD11c* (Fig 6B) expression when compared to the CHOW for both the WT and the C5aRKO, however, the C5aRKO mice on DIO had lower expression vs the WT on DIO. The immune responses of adipose tissue to LPS and C5a were evaluated. In in WT mice, LPS stimulation resulted in an increase in MCP-1 (Fig 6C) and KC (Fig 6D) secretion. In C5aRKO, there is a similar LPS stimulation of both MCP-1 and KC secretion (Fig 6C and D). In adipose tissue from WT mice, while addition of C5a alone had no effect on MCP-1 and KC secretion, C5a has a synergistic effect to LPS. By contrast, in C5aRKO mice there is no C5a effect, and no synergy to the LPS effect.

As ASP and its precursor C3 are both immune complement factors produced by adipose tissue, which affect adipose tissue energy storage, we evaluated secretion in WT and C5aRKO mice. As shown in figure 6E and 6F, C5aRKO mice on CHOW diet produced significantly less C3 and ASP, although DIO diet resulted in increases in both WT and C5aRKO mice (with a trend to less in the C5aRKO mice).

Finally, there were changes in mRNA expression in gonadal adipose tissue of C5aRKO, as compared to WT controls. As showed in figure 6G, we observed lower mRNA expression of *CD36* in C5aR mice on both diets and higher *DGAT1* expression in adipose tissue of C5aRKO mice on the CHOW diet (Fig 6G).

## Discussion

In the present study, the effects of C5aR deficiency on energy homeostasis, particularly energy expenditure, substrate utilization and storage as well as energy balance were evaluated. While it was previously shown that C5a stimulates food intake after central administration [30] our study showed no effects of C5aR deficiency in mice on food consumption whether on a CHOW or DIO diet.

On the other hand, this study demonstrates metabolic effects of a disrupted C5a-C5aR pathway: more specifically, C5aRKO mice have decreased body and adipose tissue weights and decreased fat storage whether on a CHOW or DIO diet. This is associated, for the DIO treated C5aRKO mice, with no change in food intake, but increased energy expenditure which could be centrally mediated. In C5aRKO mice, in spite of the decreased fat mass, glucose clearance and insulin tolerance are comparable to WT littermates. On the other hand, both groups of C5aRKO mice displayed lower lipid factors, including lower plasma triglyceride and NEFA and, on a DIO diet, lower cholesterol as well as a faster lipid clearance following a postprandial fat tolerance test. Lastly, our assessment of C5aR in WT mice, convey that there is a direct impact of the diet on C5aR, since we observed increased *C5aR* mRNA expression in the gonadal adipose tissue, muscle and liver tissues of the WT mice on the DIO diet. Altogether, these results suggest a re-partitioning of substrate away from storage and towards usage, potentially in such tissues as muscle and liver, although the direct impact of C5aR on metabolic pathways in cell studies remains to be evaluated.

Crosstalk between the immune system and adipose tissue is now widely accepted, and the integration of nutrient- and pathogen- sensing pathways goes far back in evolution and involves many factors including insulin, PPAR, mTOR, ER stress, TLRs, as elegantly reviewed by Hotamisligil [35]. However, the involvement of complement activation in adipose tissue changes is still relatively unexplored [4], [5], [16]. Complement proteins such as complement C3 [36], [37], adipsin [38], C3adesArg/acylation stimulating protein [39], properdin [10], factor H [40], C5L2 [23], [24] and C3aR [19] have all been implicated in lipid and glucose metabolism. The present effects on metabolism in the C5arKO mice may be the consequence of a direct role of C5aR in energy/lipid metabolism, as C5aR is expressed in adipose, muscle, liver, brain and other tissues involved in nutrient sensing and lipid metabolism, however indirect effects due to changes in immune responses cannot be ruled out.

While C5aR binds both C5a and C5adesArg, C5L2 ligand binding remains controversial: both C5a and C5adesArg bind with high affinity, yet the functional outcome (signalling vs decoy receptor) remains unclear; and some [12], [13] but not all [14], [41], [15] studies report low affinity binding of C3adesArg/ASP and C3a to C5L2. Further, expression of *C5L2* and *C5aR* can be tissue-specific and regulated differentially based on cell treatments or disease conditions as shown elsewhere [42], [43], and as shown in the present study. As well, co-localization has been demonstrated in human neutrophils, suggesting a mutual cooperation or regulation between the two receptors [44]. Further, we have recently demonstrated that C5L2-C5aR heterodimerize, and are internalized upon stimulation by ligands [45]. With regards to this, it is tempting to speculate on three models of action to explain the modified lipid metabolism observed in the present C5aRKO mice study.

Firstly, C5a or C5adesArg interaction with C5aR might have direct effects in adipose, muscle or liver (metabolic and/or immune), and the absence of C5aR could cause pathway disruption which leads to decreased adipose tissue mass and decreased levels of lipemic factors. C5a has been shown to have central effects on food intake [30], although whether this is mediated through C5aR or an alternate receptor (C5L2) is unknown. A recent study by Lim et al [31], demonstrating direct effects of C5a on glucose uptake and triglyceride synthesis in 3T3 adipocytes, effects that were blocked with the use of a specific C5aR antagonist, supports this interpretation.

A second mechanism might be that, in the absence of C5aR, there is increased interaction of C5a with C5L2 that then leads to these effects on adipose tissue/lipid metabolism. Enhancement of C5a interaction with C5L2 when C5aR is deficient or blocked with antagonists has been proposed as the mechanism for C5a effects on sepsis [46]. Evaluation of potential direct effects of C5a on adipocytes from C5aRKO mice in future studies will answer these questions.

A third mechanism would be an indirect effect of C5aR deficiency, in which the increased availability of C5a and C5adesArg, due to the absence of C5aR, interferes with ASP interaction with C5L2 pathway leading to the metabolic effects demonstrated. As ASP has been shown to stimulate triglyceride synthesis in adipocytes, an effect that is absent in adipose tissue from C5L2KO mice, this would support this mechanism. Further, a recent study demonstrating that both ASP/C3adesArg and C5a stimulate C5L2-C5aR heterodimerization in adipocytes, leading to activation of intracellular signalling pathways is supportive of this [45].

However, we cannot exclude that all three mechanisms may be involved in the phenotype identified, and we speculate that the changes in adipose tissue function could be a consequence of (i) the absence of C5a-C5aR interaction (which removes the effect of C5a on lipid storage) and (ii) increased C5a interaction with C5L2 (a consequence of higher plasma C5a, lower adipose tissue C5L2 and lower production of ASP as shown in the present study) which leads to (iii) interference of ASP-C5L2 interaction, and prevention of ASP stimulation of lipid storage On the other hand, the absence of C5aR in adipose tissue did not prevent (i) DIO-induced increase in macrophage infiltration (based on F4/80 and CD11c expression) or (ii) LPS-induced increases in MCP-1 and KC secretion, although the C5a synergy was lost in C5aRKO. Overall, these results suggest that, at least in adipose tissue, the absence of C5aR induces metabolic changes with minor changes in immune response.

The recent paper by Lim *et al* is complementary and supportive of the present study, demonstrating that *in vivo* inhibition of the C5a-C5aR pathway, using receptor-selective antagonists of C5aR, improved the presentation of metabolic syndrome in diet-induced obese rats, with less increase in body weight, visceral fat and adiposity as well as showing improvements in glucose and insulin resistance [31]. Furthermore, they showed using *in vitro* studies on 3T3-L1 adipocytes, the involvement of the C5a-C5aR pathway in promoting energy conservation by increasing glucose and fatty acid uptake while inhibiting cAMP signalling and lipolysis [31]. Their results, coupled with the present study indicating higher energy expenditure, lower adipose tissue, and enhanced fat clearance suggest that blockade of C5aR, as targeted in the development of antagonists (review: [25]), may be beneficial in unexpected ways.

### Conclusion

The present study adds to and extends the accumulating data that the complement immune system is involved in many facets of adipose tissue metabolism and establishes a foundation that C5aR, in addition to its effect on the immune system, plays a role in energy metabolism and especially on lipid metabolism.

## Supporting Information

Table S1 Sequences for primers used in RT-PCR. All sequences were obtained through the mouse primer depot resource site.
(DOCX)
