# Supplementary material for: C5a Receptor Deficiency Alters Energy Utilization and Fat Storage
Source: PLoS One. 2013 May 7;8(5):e62531. doi: 10.1371/journal.pone.0062531 (PMC3646841; doi:10.1371/journal.pone.0062531)
Supplement: Table S1 — Sequences for primers used in RT-PCR. All sequences were obtained through the mouse primer depot resource site. (DOCX) [file pone.0062531.s001.docx]

**Supplementary Table 1:** Sequences for primers used in RT-PCR. All sequences were obtained through the mouse primer depot resource site.

| Gene name | Right primer sequence | Left primer sequence |
| --- | --- | --- |
| *DGAT1* | TCACCACACACCAATTCAGG | GACGGCTACTGGGATCTGA |
| *DGAT2* | GAAGATGTCTTGGAGGGCTG | CGCAGCGAAAACAAGAATAA |
| *FAT/CD36* | CCTGCAAATGTCAGAGGAAA | GCGACATGATTAATGGCACA |
| *FAS* | CCTCAGCTTTAAACTCTCGGA | CAGACATGCTGTGGATCTGG |
| *LPL* | TGTGTCTTCAGGGGTCCTTAG | TTTGGCTCCAGAGTTTGACC |
| *PPARG* | TCTTCCATCACGGAGAGGTC | GATGCACTGCCTATGAGCAC |
| *F4/80* | CTTTGGCTATGGGCTTCCAGTC | GCAAGGAGGACAGAGTTTATCGTG |
| *CD11c* | CTGGATAGCCTTTCTTCTGCTG | GCACACTGTGTCCGAACTC |
| *C5L2 (Gpr77)* | CTGGTGGTGTGGTTCATCAT | GCTCACATCCAGGAAGCTGT |
| *Eef2* | CGGATGTTGGCTTTCTTGTC | GCTTCCCTGTTCACCTCTGA |

*C5L2*: G-protein receptor 77 (GPR77), *CD11c*: integrin chain alpha X, *DGAT1*: diacylglycerolacyltransferase-1, *DGAT2*: diacylglycerolacyltransferase-2, *Eef2*: elongation factor 2, *F4/80*: FAT/CD36: fatty acid transporter, *FAS*: fatty acid synthase, *LPL*: lipoprotein lipase, *PPARG:* peroxisome-proliferator activated receptor gamma
